# TMT-Based Quantitative Proteomic Profiling of Overwintering *Lissorhoptrus oryzophilus*

**DOI:** 10.3389/fphys.2019.01623

**Published:** 2020-01-21

**Authors:** Zhang Xinxin, Yang Shuang, Zhang Xunming, Wang Shang, Zhang Juhong, Xi Jinghui

**Affiliations:** ^1^College of Plant Sciences, Jilin University, Changchun, China; ^2^College of Life Science and Oceanography, Shenzhen University, Shenzhen, China; ^3^Key Laboratory of Optoelectronic Devices and Systems of Ministry of Education and Guangdong Province, College of Optoelectronic Engineering, Shenzhen University, Shenzhen, China

**Keywords:** insect proteomics, *Lissorhoptrus oryzophilus*, overwintering, DEP, TMT

## Abstract

Adaptations to low temperature play a critical role in restricting the geographical distribution of insects. Decreasing day lengths and temperatures trigger seasonal cold adaptations in insects. These adaptions include changes in expression at the miRNA, mRNA and protein levels. The rice water weevil (RWW), *Lissorhoptrus oryzophilus*, introduced from the Mississippi River, is a globally invasive pest of wetland rice that can survive at the northern border of China. To investigate the changes in expression at the protein level in overwintering female RWW adults, 6-plex tandem mass tags (TMTs) were used in overwintering and summer adults. By using a proteome database available for Curculionidae, 1077 proteins were quantified, 183 of which differed significantly between the overwintering and summer samples. To further understand these differentially expressed proteins (DEPs), bioinformatics analyses such as gene ontology (GO) enrichment analyses were performed. DEPs associated with the terms binding, structural molecule activity, catalytic activity, multicellular organismal process, extracellular region, chitin binding, metabolic process, intracellular part and organic cyclic compound binding were altered by selection during winter. The changes in the expression of these proteins suggest that the proteins are important for RWW survival in winter.

## Introduction

As an international quarantine species, rice water weevil (RWW; *Lissorhoptrus oryzophilus* Kuschel) causes severe economic problems in wetland rice agriculture, resulting in losses of up to 25% in untreated fields (Reay-Jones et al., 2008). The parthenogenetic female weevils invade rapidly in temperate rice growing regions around the world (Aghaee and Godfrey, 2014). In China, RWW was first identified in 1988; it is now distributed in 78% of the provinces and has become the most widespread invasive pest (Zhuo et al., 2018). This insect is located in areas with seasonal and environmental variability and must address periodic ecological adversity (Aghaee and Godfrey, 2014; Wan and Yang, 2016). Generally, RWW adults undergo prolonged exposure to subzero temperatures from October to March in the winter season in northern China, and the extremely low temperatures can even reach −30°C such as in Harbin (Li et al., 2018). To date, there has been little research on how RWW overcomes the physiological challenges in extreme winter conditions (Yang et al., 2018).

During overwintering, insects are exposed to fluctuating, freezing temperatures accompanied by adversities such as a risk of dehydration, lack of food and reduction in oxygen levels (Holmstrup et al., 2002). Among environmental factors, low temperature is a critical factor that affects the metabolic rate, distribution and ultimately the survival ability of insects (Wilches et al., 2016). As the metabolic rate of insects is temperature-dependent, low temperature causes indirect injuries such as persistent suppression of cellular metabolism, loss of ion homeostasis and accumulation of toxic metabolic end products (Dollo et al., 2010; Koštál et al., 2011; Teets and Denlinger, 2013; Sinclair, 2015). Direct injuries also lead to membrane damage, which is generally caused by phase transition, restructuring of the cytoskeleton caused by actin depolymerization and deleterious aggregations of denatured proteins. The crystallization of water also causes mechanical damage to cell membranes (Steponkus, 1984; Tursman and Duman, 1995; Feder and Hofmann, 1999; Kim et al., 2006; Teets and Denlinger, 2013).

Various aspects of the mechanism via which overwintering insects survive low temperature conditions have been studied (Rinehart et al., 2007; Clark and Worland, 2008; Fu et al., 2019; Hao et al., 2019). Insects enhance their cold resistance by means of behavioral, biochemical, and physiological strategies. In Japan, beetle larvae migrate to 10 cm below the surface of the soil, which provides a thermal buffer equivalent to moving 100 km south, and snow is another kind of thermal buffer (Sinclair et al., 2003). A variety of small molecules, such as polyhydric alcohols (glycerol, sorbitol, etc.), sugars (trehalose and glucose) and amino acids (proline and alanine) are thought to be cryoprotectants in insects (Bale and Hayward, 2010). These molecules are involved in stabilizing cell membranes and proteins, enhancing the supercooling ability of insects, and preventing infiltration damage to cells (Teets and Denlinger, 2013). Proline has been suggested to participate in the protection of membranes against dehydration during multigelation (Neufeld and Leader, 1998; Ramløv, 1999; Wharton, 2011). Unpredictable temperatures may greatly influence the cold hardiness of insects. Many freeze-avoiding insects (which die before they are frozen) have developed a rapid cold-hardening (RCH) mechanism, which instantaneously responds to severe fluctuations in temperature (Sinclair et al., 2003). Another strategy is diapause, a form of hormone-regulated dormancy that prepares the insect for expected periodic adverse conditions by stunting development and significant metabolic suppression (Lester and Irwin, 2012).

To have the minimal amount of energy needed to survive the winter and reemerge in the warm spring, overwintering insects must store enough energy prior to overwintering, as no food is available in winter (Sinclair, 2015). The main food stores are lipids, which offer the most dense energy storage. Sinclair et al. (2011) speculated that insects that do not feed during the winter rely on lipid consumption. Carbohydrates and storage proteins may also be used as fuel, and storage proteins may facilitate amino acid transfer between insect larvae and adults (Brien et al., 2002; Sinclair, 2015).

Proteomics methods have been developed successfully to investigate overwintering insects (Carrasco et al., 2011a, b; Teets and Denlinger, 2016). Tandem mass tag (TMT) technology is a powerful tool for precise and accurate quantitative proteomics. This method has been widely used to characterize protein expression profiles and investigate and compare functional changes at the protein level in vertebrates and invertebrates (Thingholm et al., 2010; Bogle et al., 2017). The 6-plex TMT method is a labeling strategy with six (126, 127, 128, 129, 130, 131) isobaric tags. Theoretically, these six labels have the same mass, and all the peptides in a sample are labeled with one chemical tag. The identical peptides from different samples produce identical precursor ions, and the same ions are used to compute quantity (Liu et al., 2016).

Using 6-plex TMT labeling-based proteomics approach, we identified and quantified differentially expressed proteins (DEPs) in overwintering and summer female RWW adults. The results offer insight into the differences in metabolic processes between the two stages and enhance our understanding of RWW overwintering diapause. The parthenogenetic female adult weevils are active in terms of feeding and reproduction in summer and can be used as control for overwintering responses. In addition, the results are crucial for predicting future outbreaks and their long-term effects.

## Materials and Methods

### Insect Collection

*Lissorhoptrus oryzophilus* female adults were collected in mid-June 2017 (summer) and mid-November 2017 (overwintering), from paddies and ridges around Changchun (43°88′N, 125°35′E), Jilin Province, China. Both samples were immediately immersed in liquid nitrogen (one insect per tube), then transported to laboratory and stored at −80°C. Local temperature was shown in Supplementary Figure S1.

### Protein Extraction

The summer and overwintering RWW (four adults and replicated three times each) were subjected to protein extraction by using the phenol extraction method, and proteins were precipitated by methanol/ammonium acetate as described previously by our laboratory (Yiou et al., 2013). The extracted samples were solubilized with lysis buffer. Protein concentrations were determined with the Modified Bradford Protein Assay Kit according to the manufacturer’s instructions (Liu et al., 2018).

### Protein Reduction, Alkylation, Isobaric Labeling, and Sample Cleanup

One hundred micrograms of each protein was treated with 1 M DL-dithiothreitol (DTT) to obtain a final concentration of 50 mM and incubated for 1 h at 37°C. Then, 1 M iodoacetamide (IAA) was added to obtain a concentration of 120–150 mM, and the mixture was incubated for 30 min at room temperature (RT) in the dark. The samples were digested with trypsin at a ratio of 1:20 for 12–16 h. Then, the pH was adjusted to 8.5 with 1 M TEAB (pH 8.5). The samples were labeled using TMT six-plex Isobaric Label Reagent Set (Thermo Fisher Scientific, San Jose, CA, United States) according to the manufacturer’s protocols. Each peptide solution was incubated for 1 h at RT and quenched for 15 min with 8 μL of 5% hydroxylamine solution in water. After labeling, the samples were desalted by a SEP-PAK Kit. Sample loading was performed by eluting cartridges with 0.6 mL of 0.1% acetonitrile-miscible trifluoro acetic acid (TFA) twice and then with 0.6 mL of 0.1% water-miscible TFA. Then the samples were eluted with 0.1% formic acid and eluted with a mixed solvent of containing 80% acetonitrile and 0.1% formic acid (1.2 mL) for three times. The sample was then dried by using a vacuum centrifuge. To reduce the complexity and improve the separation, protein characterization and confidence level of the tested sample, the lyophilized TMT-labeled peptides were divided into 12 fractions by using a high performance liquid chromatography (HPLC) system (Shimadzu). Then, fractions were dried in a vacuum centrifuge and dissolved in 1% formic acid before mass spectrometry (MS) analysis.

### Mass Spectrometry Analysis

Samples were introduced using an Easy-nLC 1000 system at a constant rate of 280 nL/min. The resulting samples were analyzed by a Q Exactive^TM^ hybrid quadrupole-Orbitrap mass spectrometer. Then the obtained peptides were loaded onto a nanospray ion (NSI) source, and tandem mass spectrometry (MS/MS) was performed in a Q Exactive spectrometer connected to an Ultra Performance LC (UPLC) system. Using normalized collision energy (NCE), the intact peptides were then selected for MS/MS with settings of 27, 30, and 33 to detect ion fragments in the Orbitrap. In an MS survey scan with a dynamic exclusion of 30.0 s, a data-dependent process was applied alternately between one MS scan and 20 MS/MS scans for the first 20 precursor ions above a threshold of 1.0E4. The fixed mass parameter was set to 100 m/z, and the electrospray voltage is 2.0 kV. Automatic gain control (AGC) was used to prevent overload of the ion trap; a total of 5E4 ions were accumulated for production of MS/MS spectra. For MS scans, the m/z scan range was set to 350–1800.

### Database Search and Protein Annotation

**FIGURE 1:**
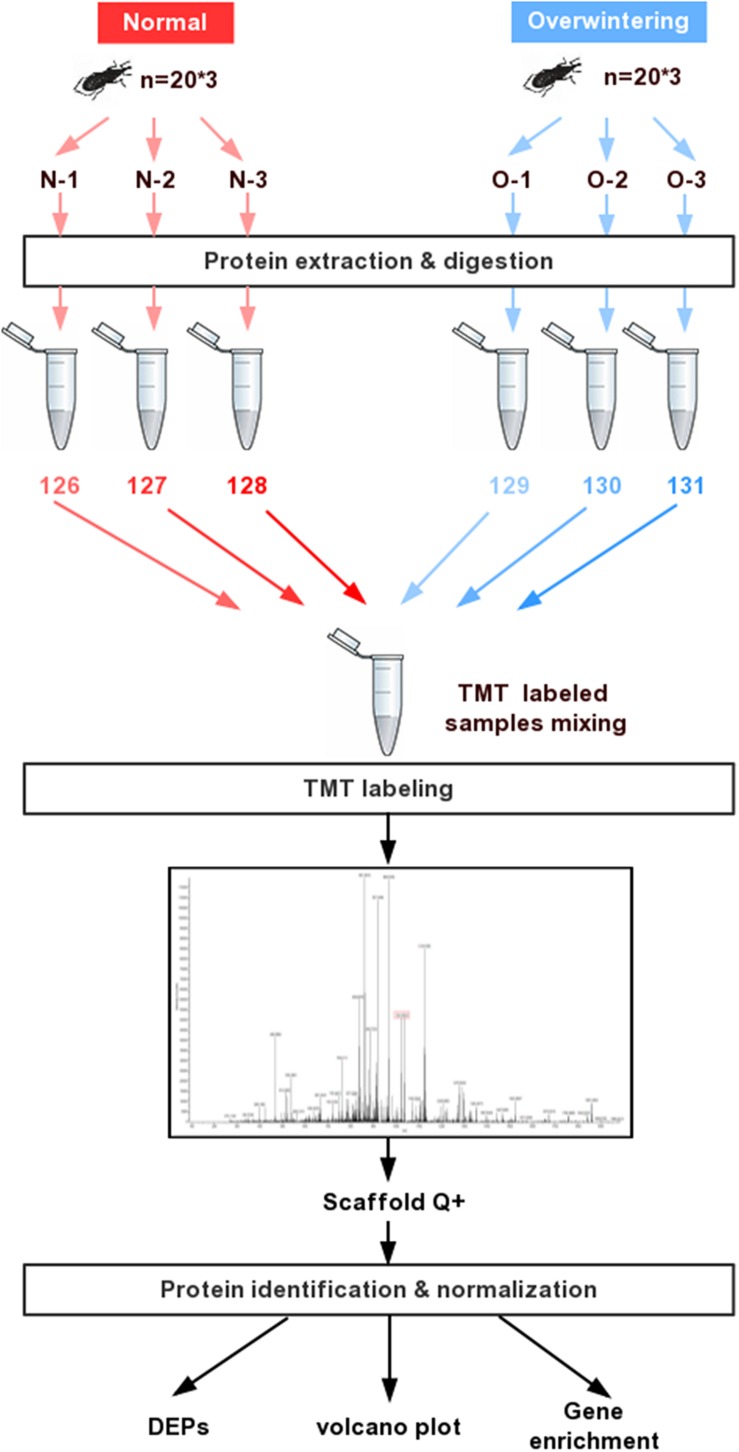
Experiment strategy for the overwintering proteomic analysis in *Lissorhoptrus oryzophilus.* The strategy mainly involved protein extraction, TMT labeling and mass spectrometry analysis.

SEQUEST was used to extract MS/MS spectra. Charge state deconvolution and isotope removal were not performed. Using SEQUEST HT, the MS/MS spectra were searched against the *Dendroctonus ponderosae* proteome database with a common contaminant list. The fragment ion mass tolerance was 0.020 Da and the parent ion tolerance was 10.0 PPM. Carbamido methylation was specified as a fixed modification. Oxidation of methionine and acetylation of the N-terminus were specified as variable modifications. The final criterion was at least two peptide matches, and the false discovery rate (FDR) threshold was specified as 0.01. All contaminant and decoy proteins were removed from the data sets before downstream analysis. The overall workflow for quantitative comparison of proteomes between the overwintering and summer samples is shown in Figure 1.

### Bioinformatics Analysis of DEPs

Using information provided by the UniProtKB/Swiss-Prot website^1^, the proteins in this study were classified according to subcellular localization and biological functions. The bioinformatics tool DAVID v6.7^2^ (Database for Annotation, Visualization and Integrated Discovery) was used to determine the gene ontology (GO) terms and identify the active biological pathways. The EASE score was used to test the gene-enrichment, and the modified Fisher’s exact *P*-value (*P* < 0.05) was significant.

### Construction of the Protein-Protein Interaction Network Among the Upregulated DEPs

The significantly upregulated proteins [fold change (FC) > 1.5] between the overwintering and summer RWW samples were selected for protein-protein interaction network construction. First, using the annotated protein names, a list of gene symbols for these proteins was retrieved from FlyBase^3^. Second, the gene symbols were transferred to GeneMANIA for network visualization with the default parameters and algorithms, and proteins that failed to be incorporated into the network were deleted (Telegina et al., 2015; Yang et al., 2018). Third, the FC level was assigned to the corresponding proteins, and the overall pathways and functional clusters were identified in Cytoscape (ver. 3.4.0).

### Content Measurement

For glycogen content measurement, each sample containing four individuals was weighed and homogenized in 1 ml of ice-cold phosphate-buffered saline (PBS) and centrifuged at 3,000 × *g* for 30 min at 4°C. The supernatants were used to estimate the glycogen content according to the manufacturer’s instructions for the Glycogen Assay Kit. For glycerol content measurement, samples were weighed and homogenized in 0.05% v/v Tween 20. Free glycerol reagent (Sigma Aldrich, Inc., St. Louis, MO, United States) was added to the experimental samples, and the samples were incubated on ice for 15 min. The liquids were then centrifuged at 16,000 × *g* for 2 min. Absorbance was tested by spectrophotometry at 540 nm and calculated by comparison with a standard curve (Boychuk et al., 2015). The results from the above experiments are reported as milligrams per gram of fresh weight (FW).

### Verification of TMT Data on Selected Candidates by *q*PCR

To obtain information complementary to the TMT results, we selected examined the expression levels of genes involved in synthesis and degradation of triglycerides and glycogen. Total RNA from the overwintering and summer RWW samples was extracted using TRIzol (TaKaRa, China). The primers used for qPCR are listed in Supplementary Table S4, and GAPDH was used as an internal reference control. The *q*PCR conditions used were as described by Wang et al. (2016), and the relative expression levels were calculated using the 2^–ΔΔ*Ct*^ method (Pfaffl, 2001). Statistical analysis was conducted by GraphPad Prism software (San Diego, CA, United States) (Wang et al., 2016).

## Results

### Identification of Proteins and Their Total and Differential Abundances

Our proteomic analysis identified 1077 proteins, which were annotated by using the *D. ponderosae* genomic information in the NCBI non-redundant (Nr) protein database (Supplementary Table S1). Of these proteins, 183 were significant DEPs with abundances that changed >1.5-fold (overwintering/summer) and *P*-values of <0.05. A total of 79 proteins were upregulated, and 104 proteins were downregulated (orange and green background colors, respectively, in Supplementary Table S1). In addition, there were 16 and 2 uncharacterized proteins among the up- and downregulated DEPs, respectively.

### Bioinformatics Analysis of DEPs Identified by TMT

**FIGURE 2:**
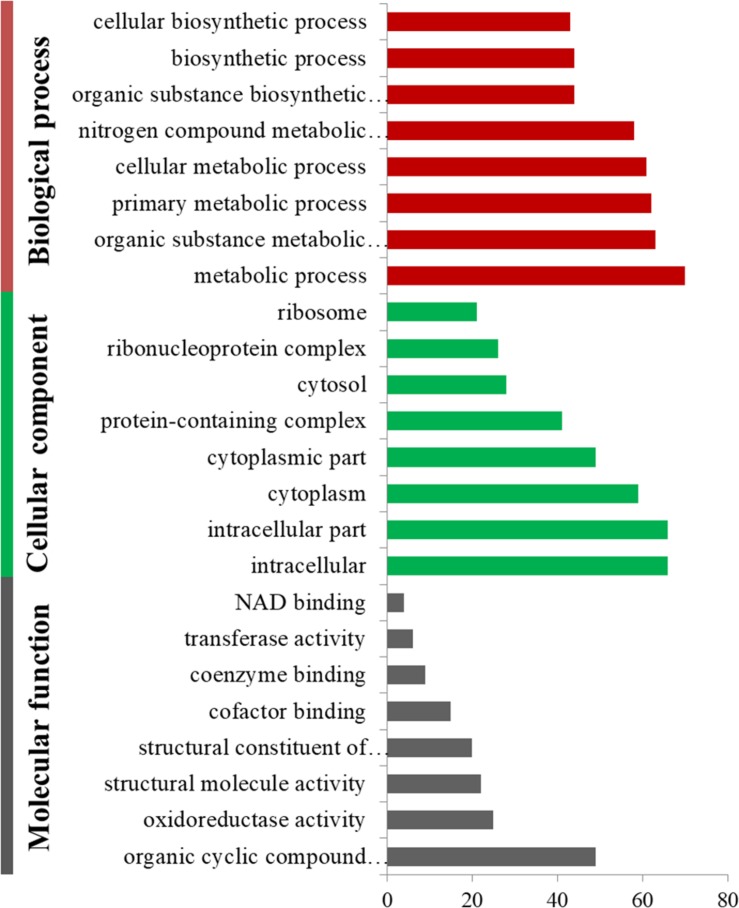
Gene ontology enrichment of downregulated differentially expressed proteins in summer and overwintering samples.

The up- and downregulated DEPs were annotated by GO with Fisher’s exact test to better understand the roles that these proteins may play in cold adaptation. The significantly up- and downregulated DEPs were classified into three categories using GO terms: biological process (BP), cellular component (CC), and molecular function (MF). The downregulated DEPs were clustered into 50 BP terms (the most representative term was “metabolic process”), 18 MF terms (the most representative term was “organic cyclic compound binding”) and 20 CC terms (the most representative term was “intracellular part”). Each of the first eight terms in BP, MF and CC determined based on *P*-values, are listed in Figure 2.

As the upregulated DEPs may play important roles in winter acclimatization, we have listed detailed information regarding these upregulated DEPs in Table 1. Surprisingly, 25 DEPs were related to the “binding” term, which suggests that these proteins have binding characteristics. The other 22 DEPs belonged to the “structural molecule activity,” “catalytic activity,” and “signaling” terms. The “metal ion binding” term contained the most (12) proteins and was related to the binding of ferric, calcium, zinc and other ions. From the proteins with significant fold change above 1.5, the one with the highest FC was “N6U3H6,” which was a “DNA-directed RNA polymerase II subunit RPB1,” with an FC of 6.36. The protein with the highest molecular weight was “N6TY73,” which was an uncharacterized protein with a “calcium ion binding” function and a molecular weight of 788 kDa.

**TABLE 1:** Detailed information regarding upregulated differentially expressed proteins in *L. oryzophilus* under overwintering conditions.

**Accession No.^a^**		**Protein name**	**Putative GO function**	**MW^c^ (kDa)**	**FC^b^**
**Metal ion binding^d^**					
N6UMB9	XP_019762844	Soma ferritin-like	Ferric iron binding	21 kDa	3.81
N6TQZ0	XP_019769531	Calsyntenin-1 isoform X1	Calcium ion binding	106 kDa	3.03
U4UDL7	XP_019754104	Uncharacterized protein	Calcium ion binding	388 kDa	1.84
J3JVB0	XP_019763879	Troponin C-like isoform X1	Calcium ion binding	26 kDa	1.59
N6TY73	XP_019766623	Uncharacterized protein	Calcium ion binding	788 kDa	1.52
N6U7C6	XP_019759314	Protein lethal(2)essential for life-like	Metal ion binding	21 kDa	2.39
J3JZK4	XP_019759081	Protein lethal(2)essential for life-like	Metal ion binding	20 kDa	2.25
J3JZ07	XP_019771215	Protein lethal(2)essential for life isoform X1	Metal ion binding	25 kDa	1.85
J3JX15	XP_019767544	LIM and SH3 domain protein Lasp	Metal ion binding	33 kDa	1.61
U4UGQ9	XP_019759575	Transcription elongation factor S-II	Zinc ion binding	34 kDa	1.75
N6URJ0	XP_019773669	Splicing factor 3A subunit 2	Zinc ion binding	31 kDa	1.60
U4UJQ7	XP_019758092	Cysteine and histidine-rich protein 1 isoform X1	Zinc ion binding	47 kDa	1.72
**Carbohydrate derivative binding**					
N6T7D8	XP_019761729	Uncharacterized protein	Chitin binding	26 kDa	4.69
N6UJI1	XP_019757077	Peritrophin-1-like isoform X2	Chitin binding	26 kDa	3.89
J3JV56	XP_019772716	Chondroitin proteoglycan-2-like	Chitin binding	29 kDa	2.93
J3JUZ8	XP_019759450	Chitinase-like protein Idgf4, partial	Chitin binding	45 kDa	1.79
N6TWD2	XP_019760659	Heat shock protein 70 A1-like	ATP binding	71 kDa	2.19
N6TZ97	XP_019769628	Ubiquitin-conjugating enzyme E2 L3	ATP binding	18 kDa	1.60
U4UE38	XP_019758794	Neutral alpha-glucosidase C-like	Carbohydrate binding	62 kDa	1.64
N6TMJ9	XP_019773075	BAG domain-containing protein Samui isoform X2	Chaperone binding	74 kDa	1.55
**Nucleic acid binding**					
N6T8 × 5	XP_019765039	hrp65 protein-like isoform X2	RNA binding	67 kDa	2.13
U4U230	XP_019768001	Sex-lethal homolog isoform X1	RNA binding	32 kDa	1.67
N6T592	XP_019763283	Serine-arginine protein 55-like isoform X1	RNA binding	36 kDa	1.61
N6TR44	XP_019770955	Serine-arginine protein 55-like	RNA binding	28 kDa	1.57
**Structural molecule activity**					
N6U3H6	XP_019761732	DNA-directed RNA polymerase II subunit RPB1	Structural constituent of cuticle	30 kDa	6.36
U4UC45	XP_019761721	Bromodomain-containing protein	Structural constituent of cuticle	57 kDa	3.58
U4U0Y9	XP_019761731	Actin cytoskeleton-regulatory complex protein PAN1	Structural constituent of cuticle	34 kDa	3.58
N6TJ67	XP_019754529	Uncharacterized protein	Structural constituent of cuticle	40 kDa	3.41
**Catalytic activity**					
N6U1M2	XP_019768132	Glucose dehydrogenase	Oxidoreductase activity	49 kDa	1.80
N6TNV8	XP_019770659	Uncharacterized protein	Oxidoreductase activity	35 kDa	1.78
U4UQF7	XP_019771283	1,5-anhydro-D-fructose reductase	Oxidoreductase activity	37 kDa	3.14
U4UI88	XP_019767072	Synaptic vesicle membrane protein VAT-1 homolog-like	Oxidoreductase activity	51 kDa	1.87
N6T3N7	XP_019768509	Ethanolamine-phosphate cytidylyltransferase isoform X1	Catalytic activity	43 kDa	1.54
**Signaling**					
U4UIY5	XP_019764323	Phosphorylated CTD-interacting factor 1 isoform X2	Wnt signaling pathway	77 kDa	1.55
N6TUG3	XP_019768959	Arrestin homolog	Signal transduction	44 kDa	1.58
N6U005	XP_019764167	Afadin-like	Signal transduction	215 kDa	1.54
**Cytoskeletal protein binding**					
U4UB92	XP_019765567	Thymosin beta isoform X10	Actin monomer binding	20 kDa	3.27
**Others**					
N6SWP2	XP_019768581	Disintegrin and metalloproteinase domain-containing protein 10	Metalloendopeptidase activity	?	5.70
U4UAR1	XP_019770374	Reversion-inducing cysteine-rich protein with Kazal motifs	Metalloendopeptidase activity	?	2.51
J3JVJ5	XP_019758050	General odorant-binding protein 56d-like	Odorant binding	17 kDa	2.69
J3JXV1	XP_019766997	Juvenile hormone epoxide hydrolase 1-like	Aromatic compound catabolic process	52 kDa	1.91
N6TPQ9	XP_019770434	β-glucuronidase	Carbohydrate metabolic process	74 kDa	1.59
U4TU44	XP_019770851	L-lactate dehydrogenase-like isoform X1	Carbohydrate metabolic process	36 kDa	1.71
N6SW87	XP_019768942	Uncharacterized protein	Integral component of membrane	155 kDa	1.85
J3JXU3	XP_019771055	Charged multivesicular body protein 4b-like	Vacuolar transport	25 kDa	2.01
N6TJ71	XP_019758650	Troponin C-like	mRNA methylation	17 kDa	1.77
U4U3I0	XP_019772090	CREB-regulated transcription coactivator 1-like	CREB transcription factor activity	54 kDa	1.72

*^*a*^UniProt and NCBI database accession numbers. ^*b*^Average ratio from three replicates by TMT experiment. A protein species was considered differentially accumulated if it exhibited a fold-change >1.5-fold (overwintering/summer) with a *P*-value < 0.05. ^*c*^Molecular weight. ^*d*^The identified proteins were sorted by molecular function terms.*

### Categorization of Differentially Downregulated Ribosomal Proteins

**FIGURE 3:**
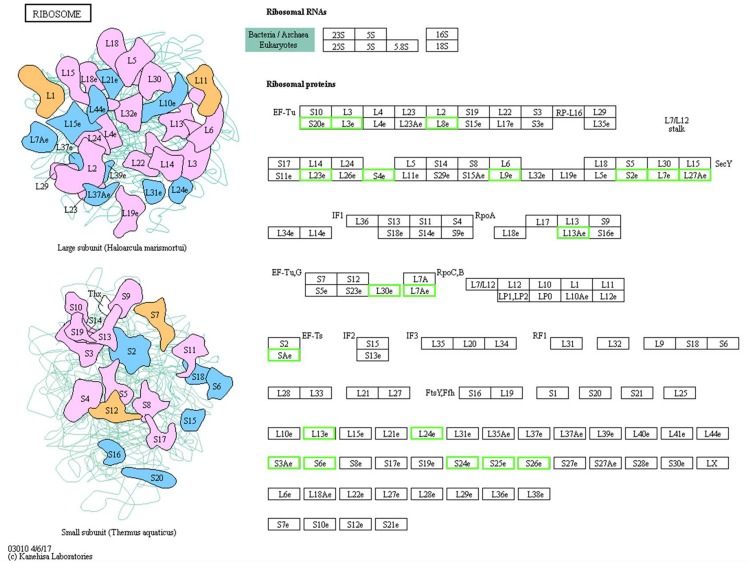
Schematic representation of the differentially expressed proteins involved in the ribosome pathway. The green boxes indicate the downregulated proteins, the specific value of the fold change is supplemented in Supplementary Table S1.

Ribosomal proteins (RPs) are known for their importance in regulating protein synthesis and maintaining the stability of ribosomal complexes. Among the 1077 quantified proteins, 44 RPs were identified including 23 40S RPs and 21 60S RPs. Of the 183 significant DEPs, 22 RPs were downregulated (9 40S and 13 60S, Figure 3). All of these RPs had a significant score based on FC, and the very large number indicates that most RPs were likely downregulated in winter, indicating that the DEPs that were RPs play important roles in RWW overwintering. The four most strongly downregulated RPs were L15, L3, L7 and L27a, which were downregulated to 0.37, 0.37, 0.41, and 0.42 times of their previous levels (Supplementary Table S1).

### Main Energy Source Metabolism Network Analysis

**FIGURE 4:**
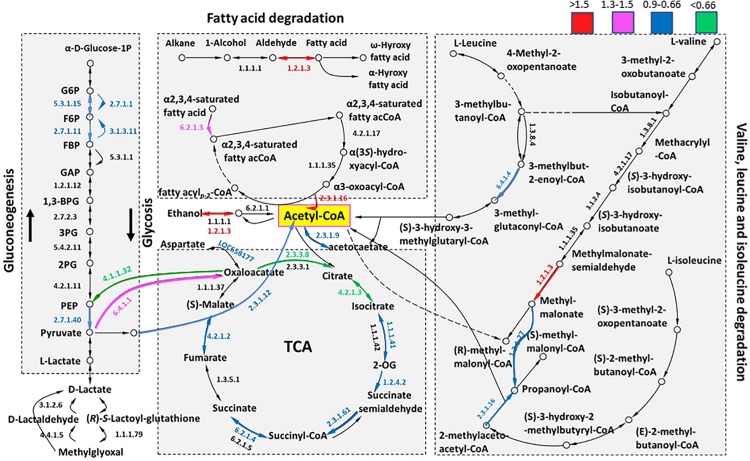
The metabolism pathway of the main energy sources. Different colors indicate the ranges of the fold change (overwintering/summer) values. The numbers indicate the EC No. of the proteins, and the color of these numbers indicates differential extent changes. The specific value of the fold change is given in Supplementary Table S2.

Herein, we constructed a metabolic network of the main energy sources, including the citrate cycle, glycolysis/gluconeogenesis, fatty acid degradation and main amino acid (valine, leucine, and isoleucine) degradation, to reveal the energy change (EC) profiles of summer and overwintering adults (Figure 4). Some of these EC profiles contained several proteins, and the preferred upregulated proteins are shown. Overall, 39 proteins were identified and quantified in the constructed energy metabolism network, and detailed information regarding all of these proteins is provided in Supplementary Table S2. In total, 28 of the proteins (71.8%) showed a downregulated trend (FC < 1), including 16 (20) TCA, 4 (7) fatty acid degradation, 5 (7) main amino acid and 9 (15) glycolysis/gluconeogenesis proteins. In contrast, pyruvate carboxylase, mitochondrial (EC 6.4.1.1), pyruvate dehydrogenase (PDH) E1 component subunit β, mitochondrial (E1β, EC 1.2.4.1), and long-chain-fatty-acid-CoA ligase 5 (EC 6.2.1.3) were slightly upregulated (1.2 < FC < 1.5, *P* < 0.05), and retinal dehydrogenase 1 (EC 1.2.1.3) was significantly upregulated (FC ≥ 1.5, *P* < 0.05). In addition, retinal dehydrogenase 1 and 3-ketoacyl-CoA thiolase participated in several metabolic processes including fatty acid degradation and degradation of valine, leucine and isoleucine. In summary, citrate cycle and glycolysis/gluconeogenesis were decreased while fatty acid degradation was activated in overwintering female RWW.

### Interaction Network of Upregulated DEPs

**FIGURE 5:**
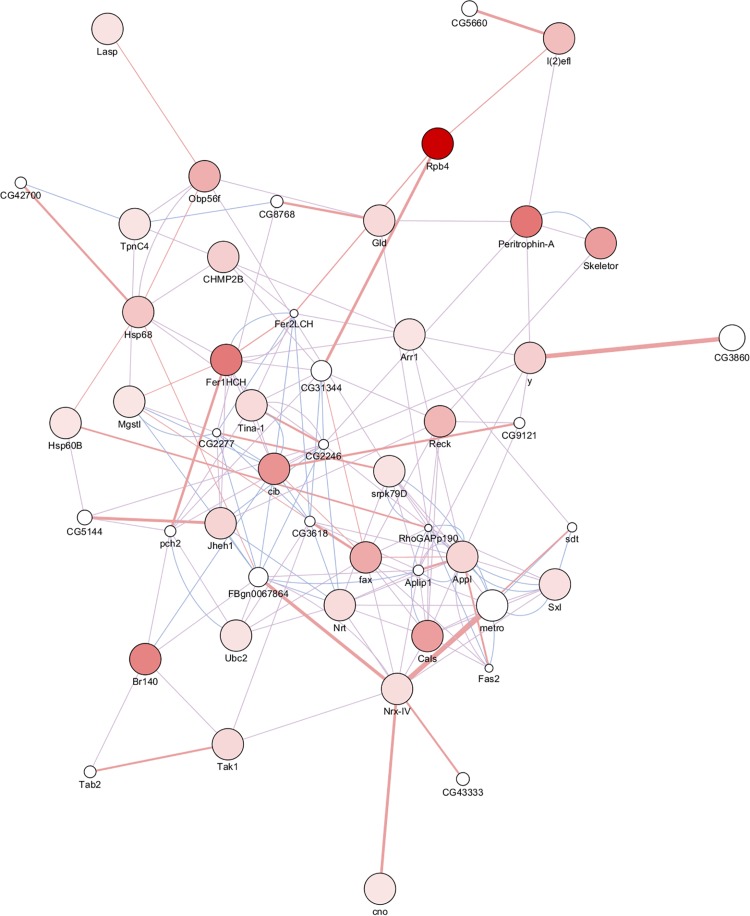
PPI network: significantly upregulated differentially expressed proteins between overwintering and summer RWW samples (Supplementary Table S3). The proteins upregulated in RWWs are colored in a gradient color from white to red, 1.52 ≤ FC ≤ 6.36.

The network contains 51 proteins, 35 of which were identified in the present study, and the remaining proteins were provided by GeneMANIA (Figure 5). Based on previous studies (Aerts et al., 2010; Guruharsha et al., 2011; Leader et al., 2018), the protein interaction networks were divided into three relation types, co-expression had the single largest share of constituents (62.46%), followed by physical interactions (35.96%) and co-localization (1.58%). Morphogenesis of embryonic epitheliumactin and cell junction were significantly overrepresented compared to the rest of the processes based on statistical results. The two proteins Fer1HCH (ferritin 1 heavy chain homolog) and Nrx-IV (neurexin IV) represented the hub with the most interactions in the network (Supplementary Table S3). Fer1HCH interacts with other proteins including cib, Jheh1, Hsp68, Arr1, Tina-1, Mgstl, and the GeneMANIA provided proteins Fer2LCH and pch2. The former proteins are associated with increased tolerance to oxidative stress and starvation. Fer2LCH provides iron nucleation sites and is essential for embryogenesis and the functions of pch2 in the monitoring the synaptonemal complex assembly and recombination during female meiosis. Up-regulation of these proteins may not only increase resistance to cold temperatures, but also be involved in parthenogenesis.

### Glycerol Production Analysis

**FIGURE 6:**
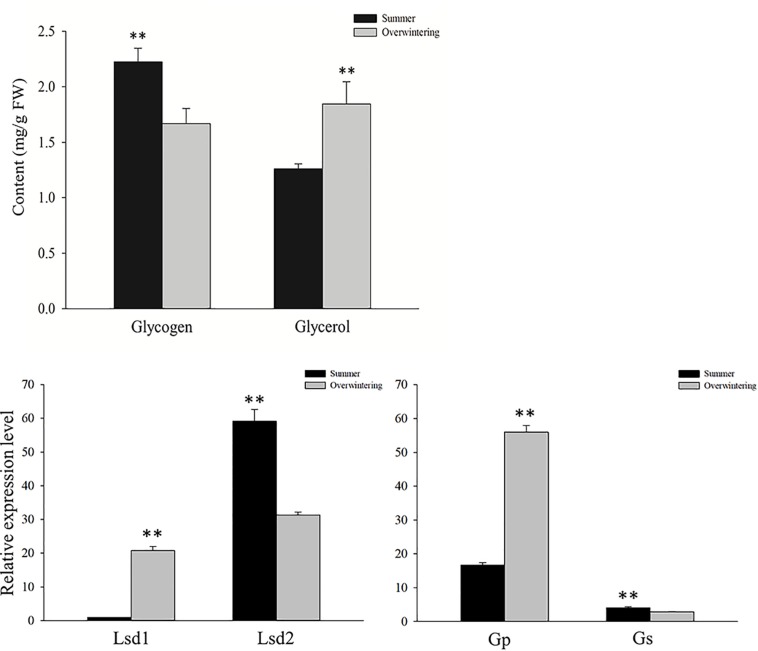
Contents of glycogen and glycerol and *q*PCR data for expression levels of genes involved in synthesis and degradation of triglycerides and glycogen between overwintering and summer RWW samples. Statistically significant differences in gene expression are indicated with asterisks: ^∗∗^*P* < 0.01.

We selectively tested the levels of glycogen and glycerol in summer and overwintering RWW female adults. Glycogen was significantly downregulated in overwintering adults while glycerol was significantly upregulated. Next, we tested the expression levels of four genes, namely, lipid storage droplet 1 (Lsd1), lipid storage droplet2 (Lsd2), glycogen phosphorylase (Gp), and glycogen synthase (Gs). The first two genes are involved in lipid storage droplet homeostasis (Teets and Denlinger, 2016; Tan et al., 2017). Lsd1 participates in the activation of triglyceride lipolysis (Fraser et al., 2017), while the role of Lsd2 is to help the accumulation of triglycerides in insect lipid droplets (Bonnett et al., 2012). The expression patterns are summarized in Figure 6. These proteins showed different expression patterns; Lsd1 was regulated in winter adults, while Lsd2 was downregulated. The latter two genes (Gp and Gs), involved in the synthesis and degradation of glycogen, also showed the same trend as Lsd1 and Lsd2.

## Discussion

Cold adaptation is defined as the process of improving cold tolerance through prolonged exposure to subzero temperatures in the field or laboratory (Teets and Denlinger, 2013). Little attention is given to how RWW cope with subzero temperatures. In this study, we intended to reveal how protein expression changes in RWW under the influence of a long-term cold period in winter through quantitative proteomics and bioinformatics.

### Energy Metabolism

Because RWWs stop feeding during the winter, their limited energy reserves must be allocated between the maintenance of basal metabolism and production of cryoprotectants. We detected 28 downregulated proteins and 11 upregulated protiens related to energy metabolism. The levels of most of the enzymes functioning in the maintenance of basal metabolism through TCA and glycolysis were decreased in overwintering adults compared to summer adults, as shown in Figure 4. Inhibition of citrate cycle activity was also observed in flesh flies, in which the levels of the intermediate products fumaric acid and citric acid were decreased during diapause (Michaud and Denlinger, 2007).

Downregulation of the key enzymes hexokinase, 6-phosphofructokinase and pyruvate kinase indicated similar results in glycolysis (Smolinski et al., 2017). Hexokinase showed a significant decrease in activity and substrate affinity under freezing conditions (Penning de Vries, 1975). Furthermore, the inhibition of phosphofructokinase at low temperatures leads to decreased glycolysis (Storey, 1987). Pyruvate kinase is a primary regulator in both glycolysis and carbohydrate metabolism. The inhibition of pyruvate kinase was accompanied by an increase in the rate of gluconeogenesis, which indicates the accumulation of glucose (Smolinski et al., 2017; Petchampai et al., 2019).

Long-chain-fatty-acid–CoA ligase 5 (ACSL5) activates free long-chain fatty acids from exogenous sources to synthesize triacylglycerol for intracellular storage and degradation via β-oxidation (Rajkumar et al., 2018). In fatty acid degradation, with a magnesium ion cofactor, ACSL5 catalyzes the conversion of a long-chain fatty acid and an acyl-CoA to the corresponding fatty acyl-CoA (Zhou et al., 2007). Upregulation of ACSL5 is a sign that the metabolic rate of fatty acid β-oxidation is increased (Rajkumar et al., 2018).

Glycerol is important for supercooling of the bodily fluids of insects, thus protecting them from low temperatures (Michaud and Denlinger, 2007). Glycerol is generally thought to accumulate from the degradation of fat or from synthetic pathways that utilize glycogen (Fraser et al., 2017). The up regulation of Lsd1 and down regulation of Lsd2 indicated that glycerol production in overwintering RWW probably occurs through a lipolytic source. The glycogen content was significantly downregulated in overwintering RWW adults, and qPCR results indicated that glycogen synthase was downregulated and glycogen phosphorylase was significantly upregulated. It was reported that fructose-1,6-diphosphatase and pyruvate kinase were inhibited during the glycerol accumulation by activation of glycogen phosphorylase (Kojić et al., 2018). The levels of both these enzymes were decreased (0.82 and 0.81) in overwintering adults, indicating that this route is also closely related to glycerol accumulation.

### RPs

At low temperatures, certain proteins are selectively degraded to meet the needs of the cell (Guy et al., 2008; Zhang et al., 2019). In the present study, 20 PRs were identified as being downregulated. RPs are responsible for ribosome biogenesis and protein translation and play important roles in controlling cell growth, division and development (Barakat et al., 2001). Studies have indicated that RPs affect fertility, viability and certain phenotypes in Drosophila (Alonso and Santaren, 2006; Paik et al., 2012). At present, there are a limited number of reports on the role of ribosome genes in stress response in plants, and few studies have been conducted on insects. The low temperature degradation of RPs suggests an adjustment of translation in cell metabolism and promotes the process of polypeptide synthesis (Carrasco et al., 2011a). The most strongly downregulated RPs in this study were the 60S RPs L15, L3, and L13 and the 40S ribosomal protein S8. A cross-species reference related to cold stress identified by bioinformatic approaches revealed the downregulation of the 3S ribosomal protein S12 and a 5S ribosomal protein in response to different cold exposures (Carrasco et al., 2011c).

The kinetic properties of RPs are different at low temperatures than at other temperatures, and a large reduction in ribosome proteins may lead to translation inefficiency. Therefore, RWW may elevate the expression of L23 and S27 to compensate for this loss or for another purpose. Upregulated RPs at low temperature may enhance proper translation or function in ribosome assembly in response to growth demands. Based on this situation, ribosomes that are more active in winter might be very important for insect overwintering.

### Structural Proteins

The levels of cytoskeletal elements, including cuticle proteins, tropomyosin, microtubule-associated proteins (MAPs), intermediate filaments, and microfilaments, were shown to be increased in insects during winter (Viswanathan and Zhu, 2002; Say, 2008; Carrasco et al., 2011b). Insects that exhibit upregulation of cytoskeletal elements during cold exposure and diapause include *Culex pipiens*, *Sarcophaga crassipalpis*, and *Aphidius colemani* (Robich et al., 2007; Yi et al., 2007; Shang et al., 2015). Upregulation of cytoskeleton-related proteins during diapause is a common response in insects, suggesting that remodeling of cytoskeletal elements plays a central role during diapause (Robich et al., 2007; Carrasco et al., 2011a).

According to the comparative proteomic analysis, MAPs showed 1.4-fold upregulation in the overwintering adults compared with summer adults. Structural MAPs are significant in the nervous system and function in both the control of microtubule cytoskeleton stability and interactions with postsynaptic proteins (Lepicard et al., 2014). Studies in *Drosophila* found that the gene *futsch*, an important gene for the whole MAP family, is involved in axon growth and neuronal development (Hummel et al., 2000), and this gene is expressed in the nervous system with the microtubule cytoskeleton at the neuromuscular junction in *Drosophila* larvae (Drewes et al., 1997).

Tropomyosin-1 is the main isoform of tropomyosin that binds and stabilizes actin cables and fibers (Singer and Shaw, 2003). This isoform plays important roles in the dynamic regulation and organization of actin filaments by inhibiting both depolymerization and polymerization of actin (Gunning et al., 2008). A previous study found elevated expression levels of tropomyosin (tropomyosin-2-like protein) in *S. crassipalpis* in response to RCH (Li and Denlinger, 2008). Furthermore, tropomyosin was shown to be important for the stable arrangement of actin filaments in *C. pipiens* at low temperatures (Kim et al., 2006; Carrasco et al., 2011b).

Increasing evidence has suggested that the levels of cuticle proteins increase in response to abiotic stress, such as in *A. colemani* during cold stress and the Colorado potato beetle, *Leptinotarsa decemlineata*, during desiccation stress (Zhang et al., 2008). These lines of evidence indicate that cuticular proteins may be involved in the prevention of cross-cuticular freezing, and may be so in RWW, representing a critical source of protection for these insects, as any ice formation outside or within the insect cuticle tends to be lethal.

### Iron-Binding Proteins

Insect neurons are protected from ions in the hemolymph by the tight septate junctions of glial cells that compose the blood–brain barrier (Stork et al., 2008). Thus, if most of the body fluid is transformed to ice, the concentrations of the remaining fluids including metal ions and other solutes are significantly increased (Storey and Storey, 2012). Ion sequestering is needed in this process. Long-term cold exposure causes cellular depolarization in insects, which is accompanied by an increase in K+ levels in the hemolymph (Overgaard and MacMillan, 2017). This would depolarize neuronal membranes, leading to initial hyperactivity followed by complete nervous system silencing. Thus, K+ homeostasis is critical for preservation of a negative resting membrane potential (Dawson et al., 1989; Fitzgerald et al., 1996). In addition, low temperature also impairs the activation of the L-type Ca^2+^ channels responsible for the action potential (AP) in larval *Drosophila* (Frolov and Singh, 2013). Cold-induced reductions in muscle AP amplitude and muscle force production seem to result from the effects of both temperature and membrane depolarization on excitation-contraction coupling (Frolov and Singh, 2013; Findsen et al., 2014, 2016).

A relationship between insect cold resistance and metal ion binding ability has been indicated (Storey and Storey, 2012). In our findings, ferritin was identified as a significantly upregulated ion binding protein, which is consistent with cold treatment studies in many insects. The ferritin level was significantly increased in *Epiblema scudderiana* after short-term cold exposure (Storey and Storey, 2010), and this protein was upregulated in response to cold in *D. ponderosae* (Bonnett et al., 2012). Transferrin was also induced by cold stress, showing up regulation at 4°C in the mulberry longhorn beetle (Sik et al., 2006). These proteins isolate free iron in bodily fluids to reduce the potential damage from the production of toxic free radicals, especially in freeze-tolerant insects with high concentrations of free metal in bodily fluids under freezing conditions (Sik et al., 2006). Transferrin is also known to be an antibiotic agent in insects which may reduce the risk of bacterial infections during the cold winter (Geiser and Winzerling, 2012). These factors may account for the multiple forms of iron-binding protein enhancement in RWW during the winter.

## Conclusion

The main objective of this 6-plex TMT proteomic experiment was successfully attained, as DEPs between female adults of overwintering and summer RWWs were found. The carbohydrate metabolism rate was depressed during the low temperate according to the TCA and glycolysis while lipid metabolism (fatty acid degradation) was increased. RWW show significantly increased levels of some proteins: ACSL5 involved in fatty acid β-oxidation; RpL23 and RpS18 involved in translation; MAPs and Tropomyosin function in microtubule cytoskeleton ferritin, involved in iron ion homeostasis. This research comprehensively identified and analyzed DEPs in overwintering and summer female RWW adults. A better understanding of the DEPs could help determine their functional roles during cold tolerance and provide new targets for bio-tech strategies development for this globally important rice pest.

## Data Availability Statement

The raw data supporting the conclusions of this article will be made available by the authors, without undue reservation, to any qualified researcher.

## Author Contributions

ZXi, YS, and XJ conceived the study. YS and ZXi undertook the experiment, analyzed the data, and drafted the manuscript. ZXu and WS provided the insects. ZJ and XJ contributed to the manuscript revisions.

## Conflict of Interest

The authors declare that the research was conducted in the absence of any commercial or financial relationships that could be construed as a potential conflict of interest.

## Footnotes

1 http://www.UniProt.org

2 http://david.abcc.ncifcrf.gov/

3 http://flybase.org/

## Supplementary Material

FIGURE S1 Field temperature during the study period.

TABLE S1 Detailed information of all identified proteins.

TABLE S2 Proteins in mainly energy source metabolism network.

TABLE S3 Interacting upregulated DEPs and their network types based on the GeneMANIA network analysis.

TABLE S4 Primers sequences used for qPCR amplification of selected genes for validation.
