# Supplementary figures and images for: TMT-Based Quantitative Proteomic Profiling of Overwintering Lissorhoptrus oryzophilus
Source: Front Physiol. 2020 Jan 21;10:1623. doi: 10.3389/fphys.2019.01623 (PMC6985562; doi:10.3389/fphys.2019.01623)

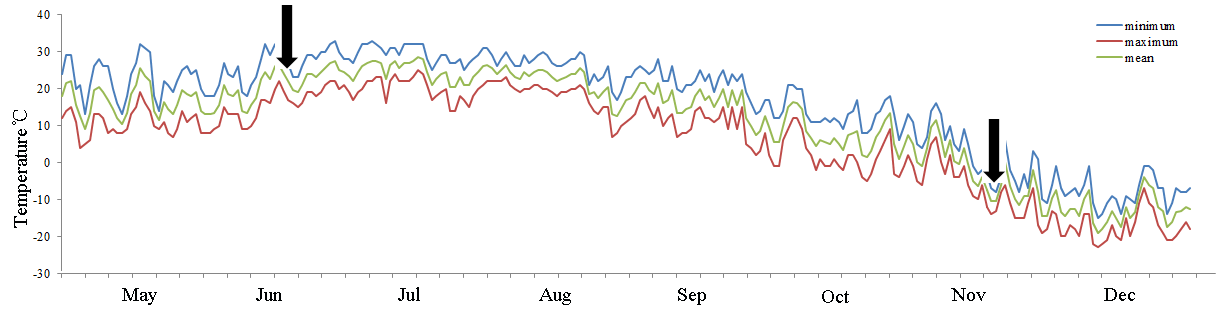

Supplement: FIGURE S1 — Field temperature during the study period. [file Image_1.TIF]
